# Validation and reliability of the translated Malay version of the psychosocial impact of dental aesthetics questionnaire for adolescents

**DOI:** 10.1186/s12955-017-0600-5

**Published:** 2017-01-26

**Authors:** Wan Nurazreena Wan Hassan, Zamros Yuzadi Mohd Yusof, Siti Safuraa Zahirah Shahidan, Siti Farhana Mohd Ali, Mohd Zambri Mohamed Makhbul

**Affiliations:** 1 Department of Paediatric Dentistry and Orthodontics, Faculty of Dentistry, University of Malaya, 50603 Kuala Lumpur, Malaysia; 2 Department of Community Oral Health and Clinical Prevention, Faculty of Dentistry, University of Malaya, Kuala Lumpur, 50603 Malaysia; 3 Faculty of Dentistry, University of Malaya, Kuala Lumpur, 50603 Malaysia; 4 Orthodontic Unit, Klinik Pergigian Cahaya Suria, Ministry of Health Malaysia, Jalan Tun Perak, Kuala Lumpur, 50050 Malaysia

**Keywords:** Malocclusion, Oral Health-Related Quality of life, Adolescent, Reliability, Validity

## Abstract

**Background:**

This paper describes the cross-cultural adaptation of the Psychosocial Impact of Dental Aesthetics Questionnaire (PIDAQ) into Malay version (Malay PIDAQ), an oral health-related quality of life (OHRQoL) instrument specific for orthodontics for Malaysian adolescents between 12 and 17 years old.

**Methods:**

The PIDAQ was cross-culturally adapted into Malay version by forward- and backward-translation processes, followed by psychometric validations. After initial investigation of the conceptual suitability of the measure for the Malaysian population, the PIDAQ was translated into Malay, pilot tested and back translated into English. Psychometric properties were examined across two age groups (319 subjects aged 12–14 and 217 subjects aged 15–17 years old) for factor structure, internal consistency, reproducibility, discriminant and construct validity, criterion validity, and assessment of floor and ceiling effects.

**Results:**

Fit indices by confirmatory factor analysis showed good fit statistics (comparative fit index = 0.936, root-mean-square error of approximation = 0.064) and invariance across age groups. Internal consistency and reproducibility tests were satisfactory (Cronbach’s α = 0.71-0.91; intra-class correlations = 0.72-0.89). Significant differences in Malay PIDAQ mean scores were observed between subjects with severe malocclusion and those with slight malocclusion based on a self-rated and an investigator-rated malocclusion index, for all subscales and all age groups (*p* < 0.05). Construct validity of the Malay PIDAQ subscales with those who rated themselves with excellent to poor dental appearance and those who felt they needed or did not need braces, showed significant associations for all age groups (*p* < 0.05). Criterion validity also showed significant association between the Malay PIDAQ scores with those with and without impact on daily activities attributed to malocclusion. There were no ceiling effects detected but floor effects were detected for the Aesthetic Concern subscale.

**Conclusion:**

The study has provided initial evidence for the validity and reliability of the Malay PIDAQ to assess the impact of malocclusion on the OHRQoL of 12–17 year old Malaysian adolescents.

## Background

Dental malocclusions comprise a wide spectrum of dental arrangements perceived as aesthetically poor, such as protruding teeth, crowded or crooked teeth, and spacing. These are often reasons for seeking orthodontic treatment among adolescents regardless of the severity of the malocclusion. In resource limited health care systems, priority is given to those with greater need for treatment. Indices such as the Index of Orthodontic Treatment Need (IOTN) provide an objective measure for classifying treatment need [1]. In Malaysia, IOTN is often used to recommend treatment under the national healthcare system. Clinical impression where even those of lower grades who were advised not to have treatment would still seek treatment at private practices suggests that there may be other underlying factors that may influence desire for treatment such as the psychosocial impact of malocclusion on their oral health-related quality of life (OHRQoL). However, this could not be measured using such previously mentioned clinical indices which were developed by expert consensus based on health, functional and aesthetic grounds.

In Malaysia, a large population study comprising a sample of 5,112 school children aged between 12–13 years old found a high prevalence of orthodontic treatment need with 47.9% school children having IOTN-DHC scores of 4 and above [2]. In a more recent study on 837 school children in Malaysia, it was found that 51.4% of 12-year-olds and 56.4% of 16-year-olds had IOTN-DHC scores of 4 and above [3]. This implies that the percentage of school children in Malaysia who require orthodontic treatment has remained high over the decade between the studies. Despite similar proportions needing orthodontic treatment, Zreaqat et al. [3] found a statistically significant higher demand for orthodontic correction among the older school children with 42.7% of 16-year-olds desiring treatment against only 22% of the younger age group (*p* < 0.001). The discrepancy in demand suggests a need to determine if patients’ desire for treatment is related to the negative impact of malocclusion on their OHRQoL and, if so, to give added support to prioritise these patients for treatment.

OHRQoL measures have gained wide interest among clinicians and researchers as instruments for evaluation of patients’ subjective interpretation of the impact of oral health on their quality of life. Malocclusion is a unique aspect of oral health in which unsatisfactory dental aesthetics are more frequently reported reasons for seeking treatment to improve patient’s well-being rather than for functional need and failure to treat would not necessarily cause physical pain, disability or become a handicap. Few instruments have been developed which include conditioned-specific impacts to assess need for orthodontic treatment [4–6]. Furthermore, any instruments used should be age appropriate as measures developed for adults may not be suitable for adolescents, who comprise the majority of orthodontic patients. The instruments also need to be validated for the specific population. Although the Oral Health Impact Profile (OHIP) [7, 8] and Child Oral Impacts on Daily Performances (Child-OIDP) index [9] have been cross-culturally adapted and validated for the Malaysian population, neither the OHIP nor Child-OIDP were specifically designed to evaluate subjective impacts due to malocclusion but were modified to allow assessment relevant to orthodontics. Thus there is a need for a condition-specific OHRQoL measure of malocclusion for the Malaysian population.

The Psychosocial Impact of Dental Aesthetics Questionnaire (PIDAQ) focuses only on the impact of dental appearance and arrangement on OHRQoL, which is the most common reason for seeking orthodontic treatment. It comprises four domains with 23 items: the Dental Self-Confidence (DSC) domain measures positive dental concept and comprises 6 items that assess dental appearance; the Social Impact (SI) domain assesses interpersonal sensitivity and comprises 8 items that measure anxiety levels towards other people’s reaction to the appearance of the subject’s teeth; the Psychological Impact domain (PI) comprises 6 items that assess negative emotions towards one’s dental appearance; and the Aesthetic Concern (AC) domain contains 3 items that assess disapproval of the image of one’s exposed dentition [10]. Three of the PIDAQ subscales were developed from scales which were able to discriminate subjects with excellent dental aesthetics and those with only minor irregularities as determined by IOTN-AC [6]: the DSC was adapted from the Self-Confidence Scale [11, 12], SI from the Social Aspect Scale of the Orthodontic Quality of Life Questionnaire (OQLQ) and AC from the Aesthetic Scale of the OQLQ [4, 5]. PI items were developed in addition to the rest of the domains [6]. The PIDAQ was specifically developed to assess perceived need for orthodontic treatment, with potential for use for assessing changes to the patient’s well-being under treatment, distinguishing patients’ and providers’ perspectives and values as well as documenting the impact of orthodontic treatment for health policy discussions and setting of clinical guidelines [6]. Originally developed in German for young adults between 18 to 30 years old [6], it has shown good cross-cultural psychometric properties [13–17]. More recently, it has been adapted for younger adolescents and in various languages [18, 19]. Klages et al. [10] have also demonstrated that the instrument adapted for German adolescents showed good psychometric properties across the range of ages 11 to 17.

Until this study, the PIDAQ has been neither translated nor validated for the Malaysian adolescent population. Thus, this study aimed to conduct a cross-cultural adaptation of PIDAQ into Malay version and assess the psychometric properties of the Malay PIDAQ for use by Malaysian adolescents.

## Methods

The cross-cultural adaptation of the PIDAQ into Malay version comprised two parts: linguistic and psychometric validations. The linguistic validation comprised the initial translation of PIDAQ into Malay, a pre-test for the Malay version, then a back translation and final review of the draft Malay PIDAQ. The psychometric validation consisted of assessing the Malay PIDAQ’s internal reliability, reproducibility, construct, criterion and discriminant validities, and floor and ceiling effects.

### Translation

The PIDAQ with 23 items comprises a positive subscale measuring the domain of DSC (6 items), and 3 negative subscales measuring the domains of SI (8 items), PI (6 items) and AC (3 items). It was originally written in German but has been published in English. In this study, both the published original and adolescent versions were reviewed. First, the PIDAQ adolescent version was translated from English into Malay by six individuals who were Malay-English bilinguals. All were of Malay ethnicity and were proficient in both languages. The group of translators comprised an expert in OHRQoL measures and questionnaire validation (ZYMY), two orthodontists (WNWH, MZMM) who were expert in the field of aesthetic dentistry, two undergraduate students (SSS, SFMA) who represented the youth perspective and a secondary school teacher (SY) who taught Malay language. The initial translations were done independently.

Following the translation process, two authors (WNWH and ZYMY) met and compared and analyzed all translations in terms of content and wording while paying attention to conceptual and item equivalence between the original index and its Malay version. Following the meeting, a single consensus translation called the draft Malay PIDAQ was agreed. All authors agreed to add a *not relevant* option to the question on *Don’t like own teeth on video* as it was felt that not all Malaysian school children have access to a video recorder (including smart phones). This was later confirmed during the pilot test.

Next, the draft Malay PIDAQ was pilot tested on 7 school children aged 12–14 years and 15 school children aged 15–17 years from the orthodontic waiting list. The pilot test was conducted by SSS and SFMA under the observation of WNWH on separate sessions for each age group. The 22 school children were asked to complete the questionnaire. The time taken to complete the questionnaire was noted to test the practicality of the questionnaire administration under fieldwork condition. Following the test, the researchers held a discussion with the schoolchildren to assess their understanding of the questionnaire’s instructions, content, answer options and wording. Words that were ambiguous were highlighted, discussed and replaced with other words with similar but clearer meanings. For example, it was found that the Malay translated verb for the word ‘self-conscious’ describing the item as *Shy because of own teeth* was hard for the participants to understand. The corresponding adult version for this item was compared and the translated Malay item was agreed to be acceptable and understood by the school children. Following the pilot test, a meeting was held among the researchers (SSS, SFMA, WNWH and ZYMM) to discuss the outcomes of the pilot test before changes to the draft Malay PIDAQ were agreed to.

The second step involved back translation of the draft Malay PIDAQ into English. This was independently carried out by the Malaysia Institute of Translation & Books. Then, the output of the back translation was thoroughly discussed (by WNWH and ZYMY), comparing the back translation with the original PIDAQ. After minor modifications, the back translation of the Malay PIDAQ was verified and the draft Malay PIDAQ was finalized.

### Psychometric validation

Subsequently, the psychometric properties of the Malay PIDAQ were tested on 12 to 17-year-old non-randomly selected participants who had not been involved in the pilot study. Sample size calculation included the consideration for detecting mis-specified factor loadings comparing the two age-groups using A-priori Sample Size Calculator for Structural Equation Models [20]. Given 4 PIDAQ subscales with 23 items, the recommended sample size for each age group at a power level of 0.80 and a probability level of 0.05 for model structure was 166 [21, 22]. This concurred with the rule-of-thumb guideline of 4 to 10 subjects per variable [23].

The questionnaire was self-administrated. Participants were classified into two age groups: 12–14 years old and 15–17 years old. For each age group, the participants completed the questionnaire either in a classroom setting or in the orthodontic clinic waiting area. Excluded were those having or having had orthodontic treatment and those with craniofacial deformities. The former were excluded to avoid confusion when the students were assessed using the Aesthetic Component of the Index of Orthodontic Treatment Need (IOTN-AC) while the latter were excluded to avoid the possibility that the psychosocial impact was due to deformity of the craniofacial features rather than due to dental aesthetics.

Participants indicated their agreement to each of the 23 items on a five-point Likert scale: *not at all*, *a little*, *somewhat*, *strongly* and *very strongly*. The responses were coded from 1 to 5 [10]. Scores for each subscale were tabulated by the sum of their items. Impact attributed to dental aesthetics was evaluated based on total PIDAQ scores, with the positive domain DSC values reverse scored. Thus, higher total scores would indicate a greater degree of negative psychosocial impact and a poorer OHRQoL accounted for by dental aesthetics [24].

Data was analyzed using IBM-SPSS-AMOS v.20 and IBM-SPSS-Statistics v.20. Chi-squared test and Fisher exact-test were used to compare equality of the proportions of the demographics between age groups while independent *t*-test was used to compare the total PIDAQ scores of the two age groups. Internal consistency was measured by confirmatory factor analysis (CFA), and Cronbach’s α for each subscale. CFA was determined by calculating estimates of the maximum likelihood discrepancy. Goodness of fit of the observed data to the model was based on a comparative fit index (CFI) ≥ 0.90 and root-mean-square error of estimation (RMSEA) < 0.08 [25]. Multiple group comparison determined measurement invariance between the two age groups. Invariance was assessed by comparing the measurement weights and the structural covariance models with the baseline unconstrained configural model. In the measurement weights model, the factor loadings were constrained equally across groups while in the structural covariance model, all estimated factor loadings as well as factor variances and covariances were constrained to be equal across groups [25]. Measurement invariance was based on a difference in CFI values at a cut-off value of 0.01 (i.e. ∆CFI > 0.01 indicates non-invariance) [25, 26]. Subscales with Cronbach’s α of between 0.70 and 0.95 were considered to have good internal consistency [27].

The PIDAQ was developed to assess treatment need in patients requesting orthodontic treatment [6]; it measures orthodontic-specific quality of life outcomes [10]. Based on a previous study [10], the discriminant validity of the Malay PIDAQ was tested by comparing its relationship with self-rated and investigator-rated perceived need for orthodontic treatment: IOTN-AC and the awareness component of the Perception of Occlusion Scale (POS). The IOTN-AC was rated using a black and white photographic 10-point-scale showing teeth with increasing severity of malocclusion [28]. The POS component comprised 6 items of malocclusion traits [29], where participants were required to evaluate each item on a 5-point Likert scale from *not at all* to *very strongly*. The self-rated and investigator-rated Malocclusion Index (MI-S and MI-D, respectively) were adapted from the study by Klages et al. [10] for analysis of the severity of malocclusion where the scores of the IOTN-AC and total scores of the POS were standardized, summed up and divided by 2 to give an index value with a 0 mean value . Four investigators (WNWH, SSZS, SFMA and MZMM) calibrated the MI-D using 40 sets of study models, assessed twice two weeks apart. The inter-operator intra-class correlation (ICC) MI-D score at T1 was 0.96 (p = 0.00; 95% CI = 0.93 to 0.97). Intra-operator ICC MI-D scores were also excellent at above 0.75 (p = 0.00) [30]: the ICC scores were 0.95 (95% CI = 0.90 to 0.97), 0.85 (95% CI = 0.71 to 0.92), 0.91 (95% CI = 0.83 to 0.95) and 0.91 (95% CI = 0.83 to 0.95). Independent *t*-test was applied to compare the relationship between the PIDAQ subscales with the malocclusion index (MI-S/MI-D) scores of those with no or slight malocclusion (lower quartiles) and those with severe malocclusion (upper quartiles).

Construct validity of the Malay PIDAQ was tested by comparing the PIDAQ with other measures measuring related constructs, i.e. perceived dental appearance rank and need for braces. Ranking of perceived dental appearance required the participants to rate their dental appearance as *excellent*, *good*, *average* or *poor*. The participants were also asked if they needed braces to correct their teeth with response options of *yes*, *no* and *don’t know*. Criterion validity was tested against the condition-specific scores of the Child Oral Impacts on Daily Performances (CS-OIDP) index [31], which measures impact of teeth on daily activities. Malocclusions were accounted for *Spaces between* and *Position of* the teeth [32]. Although *Deformity of the mouth and face* was considered as a condition-specific item, none of the participants reported any impact from this item since the study excluded those with craniofacial deformities. The CS-OIDP performance score was tabulated by adding the product of the frequency (scale from 1 to 3) multiplied by the severity (scale from 1 to 3) of the impact in any of the 8 daily activities, i.e. cleaning teeth, eating, emotional stability, smiling, speaking, relaxing, doing schoolwork and socializing. Scores were tabulated only when the impact was due to malocclusion and recorded as 0 if there was no impact due to malocclusion on the 8 daily activities. The Kruskal-Wallis test was used to assess the relationship between PIDAQ scores and perceived dental appearance rank. The Mann–Whitney statistics were used to assess the relationship between PIDAQ scores and the need for braces, and between PIDAQ scores with those who reported the presence or absence of CS-OIDP. The Pearson correlation coefficient was calculated to assess the relationship between PIDAQ scores and CS-OIDP total performance scores on the eight daily activities.

The reproducibility of the Malay PIDAQ was tested by asking approximately 30% of the subjects to re-answer the questionnaire approximately 2 weeks later. The standard error of measurement (SEM) was calculated as the square root of the residual variance of the ANOVA analysis, and the smallest detectable change (SDC) was calculated as 1.96 x √2 x SEM [27, 33]. Paired *t*-test was done to determine any significant change to the scores of the PIDAQ subscales between the first and the second test administrations. The limits of agreement was calculated as mean change ± 1.96 x standard deviation of the changes [34]. The ICC for absolute agreement by two-way random effects models was calculated, but a weighted Kappa coefficient was not determined since a weighted Kappa with quadratic weights was considered identical to ICC_agreement_ [27].

Floor and ceiling effects within each subscale were calculated as the percentage of the achieved lowest and highest possible scores. Floor or ceiling effects were considered present if the prevalence of the highest or lowest possible scores was more than 15% [27].

## Results

The participants comprised 319 and 271 subjects from the younger (12–14 years old) and older (15–17 years old) age groups, respectively. The proportions of the participants between the younger and older age groups were equally distributed and variation was not statistically significant (*p* > 0.05) by gender, source of recruitment, ethnicity and severity of malocclusion (Table 1).

**Table 1:** Demographic and clinical characteristics of the participants

Demographics	12-14 years (N)	15-17 years (N)	*p*-value
Gender			0.163
Male	137	132	
Female	182	139	
Venue			0.051
Orthodontic clinics	40	50	
Schools	279	221	
Ethnicity			0.156^c^
Malay	248	190	
Chinese	46	50	
Indian	21	28	
Others	4	3	
^a^Severity of malocclusion (Self-rated)			0.693
Lower quartile	81	66	
Middle quartile	163	133	
Upper quartile	75	72	
^b^Severity of malocclusion (Investigator-rated)			0.376
Lower quartile	86	61	
Middle quartile	152	143	
Upper quartile	81	67	

^a^Self-rated Malocclusion Index (MI-S); ^b^Investigator-rated Malocclusion Index (MI-D); ^c^Fisher exact-test, Otherwise Chi-square test

Initial analysis showed that a relatively large proportion of the participants (14.2%; *n* = 84) chose *not relevant* for the item *Don’t like own teeth on video,* which indicated that this item was relatively not relevant to their situation. Therefore, based on recommendation of handling items with large proportion of responses that could not relate the items to the participants [35] and following discussions among the authors, it was decided that the item *Don’t like own teeth on video* was removed from the AC subscale of the PIDAQ. Thus, the subsequent psychometric analyses were based on the shortened version of the Malay PIDAQ that consisted of 22 instead of 23 items.

Histogram for the younger, older and overall age groups showed normal distribution of participants’ total PIDAQ scores. Overall, the mean score was 58.0 (SD = 17.8; Range = 22–110). For the younger age group, the mean score was 58.3 (SD = 18.32; Range = 22–110) and for the older age group, the mean score was 57.4 (SD = 18.0; Range = 22–104). Independent *t*-test analysis showed that the difference between the mean PIDAQ scores of the two age groups was not statistically significant (*p* > 0.05).

The goodness-of-fit measures showed good data-fit: for both models A and B, the CFI scores were above 0.90 while the RMSEA scores were less than 0.08 with small confidence interval (Table 2). The factor loadings of Model A and that constrained for the age groups (Model B) were within the acceptable range except for the item *wish to look better* which had factor loadings that were less than 0.50. The multi-group CFA test of the constrained models with the baseline configural model showed invariance across the age groups (∆CFI < 0.01). The measurement weights model had a CFI value of 0.926 (∆CFI = 0.002) while the structural covariance model had a CFI value of 0.921 (∆CFI = 0.007).

**Table 2:** Multi-group confirmatory factor analysis showing standardized parameter estimates and fit indices

	MODEL A	MODEL B (Baseline Configural Model)	
N	590	319	271
Age-group	12–17 years	12–14 years	15–17 years
Fit Indices			
Comparative Fit Index	0.936	0.928	
Root Mean Square Error of Approximation (90% CI)	0.064 (0.059-0.070)	0.049 (0.045-0.053)	
Items in Brief	Factor loading	Factor loading	
Dental Self-Confidence			
• Proud of own teeth	0.80	0.78	0.83
• Like to show their teeth	0.56	0.54	0.61
• Pleased to see own teeth in mirror	0.86	0.84	0.88
• Teeth look nice to others	0.67	0.68	0.67
• Satisfied with own teeth’s appearance	0.79	0.79	0.77
• Find own teeth nice	0.76	0.73	0.78
Social Impact			
• Hold back their smile	0.68	0.63	0.75
• What others think	0.63	0.59	0.67
• Teasing	0.78	0.74	0.83
• People look strange at my teeth	0.78	0.80	0.77
• Shy because of own teeth	0.75	0.75	0.76
• Hiding own teeth	0.74	0.77	0.72
• Stupid comments from others	0.65	0.59	0.72
• Boys/girls find own teeth ugly	0.77	0.79	0.75
Psychological Impact			
• Envy others for their teeth	0.66	0.62	0.71
• Distressed because of others’ nice teeth	0.86	0.85	0.88
• Unhappy about own teeth	0.83	0.81	0.86
• Others have nicer teeth	0.63	0.54	0.73
• Feel bad about own teeth	0.77	0.73	0.82
• Wish to look better	0.38	0.33	0.43
Aesthetic Concern			
• Don’t like own teeth in mirror	0.75	0.74	0.77
• Don’t like own teeth on photos	0.73	0.74	0.72

Table 3 shows the internal consistency of the subscales, scale statistics and inter-item correlations of the subscales. All subscales satisfactorily achieved Cronbach’s α values of between 0.70 and 0.95. None of the inter-item correlations were ≥ 0.90 for all subscales or ≤0.30 for the DSC, SI and AC subscales. For the PI subscale, the items with inter-item correlations below 0.30 were: between *Wish to look better* and *Distressed because of others’ nice teeth* (12–14 years = 0.25; all ages = 0.29), *Unhappy about own teeth* (12–14 years = 0.23; all ages = 0.29) and *Feel bad about own teeth* (12–14 years = 0.27; all ages = 0.29). None of the item total correlations scores were < 0.30.

**Table 3:** Internal consistency, scale statistics and inter-item correlations of the Malay PIDAQ subscales

	N	Cronbach’s α	Scale statistics		Inter-item correlations			Item total correlation	
			Mean	SD	Mean	Min	Max	Min	Max
	12-14 years								
Dental Self-Confidence	319	0.87	18.73	5.61	0.53	0.35	0.69	0.49	0.77
Social Impact	319	0.88	19.40	7.30	0.50	0.31	0.69	0.56	0.75
Psychological Impact	319	0.82	17.27	5.12	0.43	0.23	0.71	0.36	0.72
Aesthetic Concern	319	0.71	4.58	2.01	0.55	0.55	0.55	0.55	0.55
	15-17 years								
Dental Self-Confidence	271	0.89	19.36	5.59	0.57	0.37	0.74	0.57	0.83
Social Impact	271	0.91	18.82	7.14	0.56	0.45	0.69	0.63	0.78
Psychological Impact	271	0.88	17.23	5.57	0.55	0.31	0.76	0.45	0.79
Aesthetic Concern	271	0.71	4.74	1.91	0.56	0.56	0.56	0.56	0.56
	12-17 years								
Dental Self-Confidence	590	0.88	19.02	5.61	0.55	0.36	0.71	0.53	0.80
Social Impact	590	0.89	19.13	7.23	0.52	0.39	0.68	0.59	0.75
Psychological Impact	590	0.85	17.25	5.33	0.48	0.29	0.73	0.40	0.75
Aesthetic Concern	590	0.71	4.65	1.97	0.55	0.55	0.55	0.55	0.55

*SD* standard deviation

Table 4 shows there were statistically significant differences in Malay PIDAQ mean scores between adolescents who rated themselves (MI-S) with no or slight malocclusion and those with severe malocclusions for all subscales in all age groups (*p* < 0.01). Similarly, statistically significant differences were observed in mean scores between adolescents who were rated by the investigators (MI-D) with no or slight malocclusion and those with severe malocclusions for all subscales in all age groups (*p* < 0.01). In all the three age groups, comparison between MI-S and MI-D showed that DSC mean scores reduced with increasing severity of the malocclusion. In contrast, SI, PI and AC subscale mean scores increased with increasing severity of malocclusion.

**Table 4:** Discriminant validity of the Malay PIDAQ with self-rated (MI-S) and interviewer-rated (MI-D) malocclusions

Self-rated Index (MI-S)												
*Age group*	*12-14 years*				*15-17 years*				*12-17 years*			
Quartile (Self-perceived malocclusion level)	Lower (Slight)	Upper (Severe)	Effect size	*p* value	Lower (Slight)	Upper (Severe)	Effect size	p value	Lower (Slight)	Upper (Severe)	Effect size	*p* value
n	81	77			66	63			147	147		
	Mean (SD)	Mean (SD)			Mean (SD)	Mean (SD)			Mean (SD)	Mean (SD)		
Dental Self-Confidence	22.65 (4.88)	13.97 (4.48)	1.86	0.00*	23.92 (4.54)	15.17 (4.32)	1.99	0.00*	23.22 (4.76)	14.65 (4.50)	1.86	0.00*
Social Impact	15.84 (6.64)	24.51 (7.45)	−1.24	0.00*	13.83 (4.93)	24.41 (7.13)	−1.88	0.00*	14.94 (6.00)	24.35 (7.32)	−1.44	0.00*
Psychological Impact	14.32 (4.47)	21.00 (4.86)	−1.41	0.00*	13.50 (4.44)	21.92 (4.98)	−1.80	0.00*	13.95 (4.57)	21.29 (4.92)	−1.55	0.00*
Aesthetic Concern	3.35 (1.51)	6.09 (2.08)	−1.61	0.00*	3.35 (1.28)	6.25 (1.93)	−1.94	0.00*	3.35 (1.41)	6.10 (1.99)	−1.69	0.00*
Investigator-rated Index (MI-D)												
*Age group*	*12-14 years*				*15-17 years*				*12-17 years*			
Quartile (Investigator-rated malocclusion level)	Lower (Slight)	Upper (Severe)	Effect size	*p* value	Lower (Slight)	Upper (Severe)	Effect size	p value	Lower (Slight)	Upper (Severe)	Effect size	*p* value
n	75	81			63	67			147	148		
	Mean (SD)	Mean (SD)			Mean (SD)	Mean (SD)			Mean (SD)	Mean (SD)		
Dental Self-Confidence	25.65 (5.14)	16.72 (5.23)	0.96	0.00*	21.56 (5.49)	17.10 (5.86)	0.79	0.00*	21.71 (5.04)	16.89 (5.51)	0.92	0.00*
Social Impact	17.19 (6.65)	20.72 (7.27)	−0.51	0.00*	16.48 (6.36)	22.60 (7.96)	−0.87	0.00*	16.63 (6.32)	21.57 (7.62)	−0.72	0.00*
Psychological Impact	15.92 (4.76)	18.33 (4.91)	−0.50	0.00*	15.02 (4.98)	19.54 (6.03)	−0.83	0.00*	15.43 (4.64)	18.88 (5.46)	−0.69	0.00*
Aesthetic Concern	3.72 (1.66)	5.19 (1.92)	−0.82	0.00*	4.11 (1.64)	5.52 (2.08)	−0.77	0.00*	3.84 (1.59)	5.34 (2.00)	−0.85	0.00*

*SD* standard deviation

*p < 0.05

Self-endorsement of the participants’ dental appearance showed statistically significant associations between Malay PIDAQ subscales and self-perceived dental appearance (*p* < 0.01) for all age groups (Table 5). For the DSC subscale, the mean scores gradually decreased as participants rated their teeth from excellent to poor. The trend was statistically significant. Conversely, the trend in PIDAQ mean scores increased in SI, PI, and AC subscales for all age groups as participants rated their teeth from excellent to poor. The trend was statistically significant.

**Table 5:** Construct validity of Malay PIDAQ with regards to ranking of self-perceived dental appearance

PIDAQ variables	Rate appearance	N	PIDAQ scores					*p* value
			Mean	SD	Quartiles			
					Lower	Middle	Upper	
*12-14 years*								
Dental Self-Confidence	Excellent	40	22.88	5.11	20.00	24.00	26.75	0.00*
	Good	156	21.06	4.34	18.00	21.00	24.00	
	Average	94	15.49	3.78	12.75	15.00	17.00	
	Poor	29	10.97	4.19	8.00	10.00	13.00	
Social Impact	Excellent	40	14.73	5.68	10.00	14.00	18.00	0.00*
	Good	156	17.59	5.75	13.00	17.00	21.75	
	Average	94	21.34	6.83	16.00	21.00	25.25	
	Poor	29	29.28	7.75	25.50	29.00	35.50	
Psychological Impact	Excellent	40	13.40	3.52	11.00	13.00	16.00	0.00*
	Good	156	15.96	4.01	13.00	16.00	19.00	
	Average	94	18.55	4.43	15.00	18.00	21.00	
	Poor	29	25.52	4.41	24.00	25.00	29.00	
Aesthetic Concern	Excellent	40	3.70	1.68	2.25	3.00	4.00	0.00*
	Good	156	3.90	1.60	3.00	4.00	5.00	
	Average	94	5.18	1.73	4.00	5.00	6.00	
	Poor	29	7.52	2.01	6.50	8.00	9.00	
*15-17 years*								
Dental Self-Confidence	Excellent	37	25.00	5.76	23.00	27.00	29.00	0.00*
	Good	127	21.29	4.19	18.00	21.00	25.00	
	Average	88	15.76	3.08	14.00	15.50	18.00	
	Poor	19	12.05	4.56	8.00	12.00	16.00	
Social Impact	Excellent	37	14.84	6.47	9.00	15.00	18.00	0.00*
	Good	127	16.26	5.25	12.00	16.00	19.00	
	Average	88	21.80	6.19	17.00	21.00	26.00	
	Poor	19	29.89	7.10	28.00	32.00	33.00	
Psychological Impact	Excellent	37	13.24	4.97	10.00	12.00	16.25	0.00*
	Good	127	15.12	3.96	12.00	15.00	18.00	
	Average	88	20.20	4.52	16.00	20.00	23.75	
	Poor	19	25.26	5.59	25.00	26.00	29.00	
Aesthetic Concern	Excellent	37	3.46	1.63	2.00	3.00	4.00	0.00*
	Good	127	4.02	1.48	3.00	4.00	5.00	
	Average	88	5.72	1.46	5.00	6.00	7.00	
	Poor	19	7.47	2.12	6.00	7.00	10.00	
*12-17 years*								
Dental Self-Confidence	Excellent	77	23.90	5.50	21.00	25.00	28.00	0.00*
	Good	283	21.17	4.27	18.00	21.00	25.00	
	Average	182	15.62	3.45	13.00	15.00	18.00	
	Poor	48	11.40	4.33	8.00	11.50	13.00	
Social Impact	Excellent	77	14.78	6.03	10.00	15.00	18.00	0.00*
	Good	283	16.99	5.56	13.00	17.00	21.00	
	Average	182	21.56	6.51	17.00	21.00	26.00	
	Poor	48	29.52	7.43	26.00	30.00	34.75	
Psychological Impact	Excellent	77	13.32	4.25	10.00	13.00	16.00	0.00*
	Good	283	15.58	4.00	13.00	16.00	18.00	
	Average	182	19.35	4.54	16.00	19.00	22.00	
	Poor	48	25.42	4.86	24.00	26.00	29.00	
Aesthetic Concern	Excellent	77	3.58	1.65	2.00	3.00	4.00	0.00*
	Good	283	3.96	1.55	3.00	4.00	5.00	
	Average	182	5.43	1.63	4.00	5.00	6.00	
	Poor	48	7.50	2.03	6.00	8.00	9.00	

*SD* standard deviation

*p < 0.05

The associations between self-perceived need for braces and PIDAQ subscales for all age groups were statistically significant (Table 6). Those who perceived that they needed braces had significantly lower DSC subscale mean scores and significantly higher SI, PI and AC subscale mean scores than did those who perceived that they did not need braces.

**Table 6:** Construct validity of Malay PIDAQ with regards to self-perceived need for dental correction

PIDAQ variables	Need braces	N	PIDAQ scores					*p* value
			Mean	SD	Quartiles			
					Lower	Middle	Upper	
*12-14 years*								
Dental Self-Confidence	No	111	21.24	4.30	19.00	22.00	26.00	0.00*
	Yes	128	15.73	5.41	12.00	15.00	19.75	
Social Impact	No	111	16.50	5.36	12.00	16.00	20.00	0.00*
	Yes	128	22.21	8.02	16.00	21.50	28.00	
Psychological Impact	No	111	14.77	3.59	12.00	15.00	17.00	0.00*
	Yes	128	19.33	5.75	15.00	19.50	24.00	
Aesthetic Concern	No	111	3.78	1.55	3.00	4.00	4.00	0.01*
	Yes	128	5.45	2.21	4.00	5.00	7.00	
*15-17 years*								
Dental Self-Confidence	No	89	22.52	4.48	9.00	20.00	26.00	0.00*
	Yes	119	15.95	4.55	13.00	16.00	19.00	
Social Impact	No	89	15.53	5.38	11.00	15.00	19.00	0.00*
	Yes	119	22.47	7.39	17.00	23.00	29.00	
Psychological Impact	No	89	14.18	4.06	11.50	14.00	17.00	0.00*
	Yes	119	20.45	5.37	16.00	21.00	25.00	
Aesthetic Concern	No	89	3.80	1.55	2.00	4.00	5.00	0.00*
	Yes	119	5.70	1.93	4.00	6.00	7.00	
*12-17 years*								
Dental Self-Confidence	No	200	21.81	4.42	18.00	22.00	25.00	0.00*
	Yes	247	15.83	5.00	12.00	15.00	19.00	
Social Impact	No	200	16.07	5.38	12.00	16.00	20.00	0.00*
	Yes	247	22.34	7.71	17.00	22.00	28.00	
Psychological Impact	No	200	14.51	3.81	12.00	15.00	17.00	0.00*
	Yes	247	19.87	5.58	16.00	20.00	24.00	
Aesthetic Concern	No	200	3.79	1.55	3.00	4.00	5.00	0.00*
	Yes	247	5.57	2.08	4.00	5.00	7.00	

*SD* standard deviation

*p < 0.05; Excluded those who answered ‘don’t know’

Table 7 shows the association between the presence of CS-OIDP impact and Malay PIDAQ subscales. Those with CS-OIDP impacts had significantly lower DSC subscale mean scores and significantly higher SI, PI and AC subscale mean scores than those without CS-OIDP impacts. The trend was statistically significant for all age groups (*p* < 0.01).

**Table 7:** Criterion validity of Malay PIDAQ with impact on daily activities attributed to malocclusion

			Mann Whitney U						Pearson correlation	
PIDAQ variables	CS-OIDP prevalence	N	PIDAQ scores					*p* value	CS-OIDP performance score	*p* value
			Mean	SD	Quartiles					
					Lower	Middle	Upper			
*12-14 years*		*319*								
Dental Self-Confidence	No	244	19.99	5.09	16.00	20.00	24.00	0.00*	−0.39	0.00*
	Yes	75	14.64	5.32	11.00	14.00	18.00			
Social Impact	No	244	18.03	6.34	13.00	17.00	22.00	0.00*	0.46	0.00*
	Yes	75	23.85	8.42	17.00	24.00	29.00			
Psychological Impact	No	244	16.28	4.43	13.00	16.00	19.00	0.00*	0.43	0.00*
	Yes	75	20.49	5.87	16.00	20.00	25.00			
Aesthetic Concern	No	244	4.14	1.73	3.00	4.00	5.00	0.00*	0.42	0.00*
	Yes	75	6.04	2.19	4.00	6.00	8.00			
*15-17 years*		*271*								
Dental Self-Confidence	No	203	20.79	5.18	17.00	21.00	25.00	0.00*	−0.42	0.00*
	Yes	68	15.19	4.69	12.00	14.50	18.00			
Social Impact	No	203	16.94	6.11	13.00	16.00	20.00	0.00*	0.53	0.00*
	Yes	68	24.44	7.06	19.00	24.00	30.75			
Psychological Impact	No	203	15.65	4.87	12.00	15.00	18.00	0.00*	0.51	0.00*
	Yes	68	21.94	4.81	18.00	22.00	26.00			
Aesthetic Concern	No	203	4.22	1.64	3.00	4.00	5.00	0.00*	0.46	0.00*
	Yes	68	6.28	1.84	5.00	6.00	7.75			
*12-17 years*		*590*								
Dental Self-Confidence	No	447	20.35	5.14	16.00	20.00	25.00	0.00*	−0.40	0.00*
	Yes	143	14.90	5.02	12.00	14.00	18.00			
Social Impact	No	447	17.53	6.25	13.00	17.00	21.00	0.00*	0.49	0.00*
	Yes	143	24.13	7.78	18.00	24.00	30.00			
Psychological Impact	No	447	15.99	4.62	13.00	16.00	19.00	0.00*	0.47	0.00*
	Yes	143	21.18	5.42	17.00	22.00	26.00			
Aesthetic Concern	No	447	4.17	1.69	3.00	4.00	5.00	0.01*	0.43	0.00*
	Yes	143	6.15	2.03	5.00	6.00	8.00			

*CS-OIDP* condition-specific child oral impacts on daily performances, *SD* standard deviation

*p < 0.05

Analysis using Pearson correlation coefficient showed that the CS-OIDP performance scores had a weak statistically significant negative correlation with DSC subscale mean scores for all age groups (Table 7). Conversely, the CS-OIDP performance scores showed a weak to moderate statistically significant positive correlation with SI, PI and AC subscale scores for all age groups.

Table 8 shows the findings of the Malay PIDAQ reproducibility test. The ICC values were above 0.70 (*p* < 0.05) for all subscales in all age groups. No statistically significant differences were found between the first and second test administration except for the PI subscale in the younger age group. The smallest detectable change (SDC) seemed to be higher in the younger age group for all subscales. Bland and Altman analysis showed that more than 90% of the scores of repeated measurements were within the limits of agreement except for AC subscale for the younger age group at 78.6%.

**Table 8:** Tests of reproducibility for the Malay PIDAQ

	ICC_agreement_			Paired *t*-test	Bland and Altman		
	(95% CI)	SEM	SDC	M_Diff_ (SD)	95% Limits of agreement		
					Lower	Upper	% Within limits
12-14 years (*N* = 103)							
Dental Self-Confidence	0.86 (0.79-0.90)*	2.76	7.65	0.03 (3.90)	−7.62	7.68	95.1
Social Impact	0.72 (0.59-0.81)*	5.06	14.03	1.39 (7.16)	−12.64	15.42	93.2
Psychological Impact	0.78 (0.68-0.85)*	3.15	8.73	1.08* (4.46)	−7.65	9.81	95.1
Aesthetic Concern	0.77 (0.66-0.85)*	1.27	3.51	−0.03 (1.79)	−3.54	3.48	78.6
15-17 years (*N* = 83)							
Dental Self-Confidence	0.89 (0.83-0.93)*	2.51	6.96	−0.84 (3.55)	−7.80	6.12	95.2
Social Impact	0.85 (0.76-0.90)*	3.70	10.26	−1.81 (5.23)	−12.07	8.45	94.0
Psychological Impact	0.85 (0.76 - 0.90)*	2.70	7.48	0.00 (3.83)	−7.48	7.48	92.8
Aesthetic Concern	0.82 (0.72 - 0.88)*	1.11	3.09	0.13 (1.58)	−2.96	3.22	94.0
12-17 years (*N* = 186)							
Dental Self-Confidence	0.88 (0.84 – 0.91)*	2.64	7.33	−0.02 (3.74)	−7.35	7.31	95.2
Social Impact	0.79 (0.72 – 0.84)*	4.53	12.54	0.69 (6.40)	−11.86	13.23	94.1
Psychological Impact	0.81 (0.75 – 0.86)*	2.98	8.26	0.60 (4.21)	−7.66	8.85	100
Aesthetic Concern	0.80 (0.73 – 0.85)*	1.20	3.32	0.04 (1.70)	−3.28	3.37	93.5

*CI* confidence interval, *SEM* standard error of measurement, *SDC* smallest detectable change, *M*_*Diff*_ mean differences, *SD* standard deviation

*p < 0.05

None of floor and ceiling effects for all subscales were above the cut-off value of 15% in all age groups except for the AC subscale for the younger age group and overall age group (Table 9). AC subscale had the highest prevalence of the floor effects of between 14.8%-16.3% in all age groups.

**Table 9:** Floor and ceiling effects of the Malay PIDAQ

	12-14 years (*N* = 319)		15-17 years (*N* = 271)		12-17 years (*N* = 590)	
	% Floor	% Ceiling	% Floor	% Ceiling	% Floor	% Ceiling
Dental Self-Confidence	1.9	1.3	1.1	3.3	1.5	2.2
Social Impact	3.4	0.6	5.5	0.4	4.4	0.5
Psychological Impact	0.9	2.5	1.8	1.8	1.4	2.2
Aesthetic Concern	16.3	2.2	14.8	2.2	15.6	2.2

## Discussion

The cross-cultural adaptation of the Malay PIDAQ based its protocols on those of previous studies [9, 10, 19] and on OHRQoL expert advice and recommendations [27, 36]. Herdman et al. [36] outlined 6 steps for cross-cultural adaptation of an index, which are conceptual, item, semantic, operational, measurement and functional equivalences. In brief, conceptual equivalence ascertains that the answers to each question reflect the same concept so that they are meaningful in both cultures and languages concerned. Item equivalence is concerned that the item estimates the same parameters on the latent trait being measured. Semantic equivalence establishes that the meaning of the item is equally maintained after translation. Operational equivalence is concerned with the possibility to use similar format, instructions, mode of administration and measuring methods, while measurement equivalence refers to the achievement of acceptable similar psychometric properties. Functional equivalence is the extent to which the instrument does what it is supposed to do equally well in two or more cultures and is considered achieved when all other types of equivalence in the model have been achieved.

The sample age range included the expected age of the 5-year-period of secondary school children in Malaysia from Form 1 (12–13 years old) to Form 5 (16–17 years old). The age range was similar to those commonly seen to request for orthodontic treatment at the Malaysia Ministry of Health orthodontic clinics. Unless early interceptive treatment is required, referrals to orthodontic specialists by dental officers will be done only when all permanent teeth have erupted, which is usually by around 12 years of age. At these government-sponsored institutions, treatment is offered free of charge to children whose parents are in the government service or at very minimal fees to those whose parents don’t work for the government. The considerably lower cost for treatment at these clinics compared with that in private practices is a major reason for the high treatment demand at these institutions. Due to limited resources, treatment at these institutions is offered only to schoolchildren and adults who require multidisciplinary management. Therefore, this study focuses on this adolescent age group where evidence of impact on their OHRQoL may be of interest to national health policy makers in determining services offered to them. Klages et al. [10] has advocated controlling the quality of the test for the validity of the PIDAQ for adolescents by narrowing the age range of the study age groups. Division of the age range for this study was influenced by the Ministry of Education’s policy that does not permit data collection from students taking national examinations, that is, the Form 3 and Form 5 classes, which are usually composed of 15- and 17-year-old schoolchildren, respectively. Thus participants from schools were mainly recruited from the Forms 1 and 2 classes who represented the younger age group and from Form 4 students who represented the older age group, while participants from orthodontic clinics included all target ages who attended to request orthodontic treatment.

Conceptual, item and semantic equivalences of the Malay PIDAQ were established through discussions and advice by the expert in OHRQoL throughout the linguistic validation process. Conceptual equivalence was based on the literature of the impact of malocclusion on the OHRQoL of Malaysian youths based on studies that used the OHIP-14 [37] and CS-OIDP [38] and on discussions between experts and a sample of the target population. In addition to information gained from the local professional literature, discussions with orthodontists and participants in the pilot study provided appropriate conceptual evidence of the impact of dental aesthetics on the local population. Item equivalence was established by identifying that all items were relevant to the population apart from one item (*Don’t like own teeth on video*) which was initially maintained in the scale but the response format was amended. Semantic equivalence was determined initially by forward translation to identify words that were difficult to translate or to be understood by the schoolchildren such as *self-conscious* and *upset*. In such situations, the original and adolescent versions were compared, and choice of the most appropriate words was made by discussions and consensus with experts and confirmed by pilot testing. This process was followed by back translation by an independent professional translator not involved in the study to avoid bias.

Operational equivalence in terms of questionnaire format, response options and method of administration was established. Pilot testing showed the format to be acceptable to the participants. In terms of measurement methods, as expected due to limited access to video recording devices among schoolchildren, the item *Don’t like own teeth on video* was not universally relevant. Participants in the pilot test and a relatively large proportion of those from the psychometric validation (14.2%) confirmed that this item was irrelevant to them. Although modification to the response format for this item by adding the answer option *not relevant* for the Malay PIDAQ was initially considered to maintain the item in the index, having large proportion of *not relevant* responses may have implications to future studies. Such responses may not be a concern in longitudinal studies that compare within-subject change but may be an issue in studies that compare differences between groups [35]. Jokovic et al. [35] suggested 3 methods to handle such situations: (1) to exclude cases with such responses. Excluding cases with such responses may result in loss of valuable information from participants who did not have opportunity related to the activity of only 1 item of the instrument; (2) to modify the scores. Modifying scores of not relevant responses using varying methods of imputation can affect the precision and accuracy of an instrument; or (3) to remove the item. Jokovic et al. [35] suggested that an item could be removed from the instrument that has a large proportion of responses that were not applicable to the participant. The percentage was not specified although another study have recommended that items with more than 10% responses in such category may not be suitable to be included in an item bank [39]. Thus, after discussion among the authors, it was decided to remove the item from the Malay PIDAQ. In terms of response options, generally, the 5-point Likert scale was a suitable method to elicit response since the entire spectrum of responses was used in responding to all items with varying frequency. Other modes of administration apart from self-administered questionnaire were limited to the pilot test, where each item was discussed one by one to determine their understanding of each item. In designing this study, the questionnaire was intended to be used in a large linguistically mixed sample population study or for any young Malaysian participants to answer in the waiting room in a busy orthodontic clinic since the literacy rate for basic Malay is high (95.2%) among secondary school children in Malaysia [40]. Telephone surveys were considered impractical since telephones or mobile phones are considered a luxury item and ownership of them is limited among adolescents in Malaysia. In addition, face-to-face interviews and mail correspondence by Malaysians have shown poor response rate (56.8% and 48.1%) [8].

Measurement equivalence was assessed through psychometric validation by tests of reliability and internal consistency, test of reproducibility and tests of construct validity [36] and by comparing the results with previous study on German adolescents population [10]. Test of responsiveness was not done and is recommended for longitudinal validation of this measure.

The multidimensional construct structure of the Malay PIDAQ for Malaysian adolescents was supported by good data-fit results and was invariant across the age groups. Measurement invariance across age groups was based on the ∆CFI rather than the commonly used ∆*χ* ^2^ since ∆CFI as a test of invariance is not affected by sample size unlike ∆*χ* ^2^ [26]. This study also did not use the fit-statistics like a previous study that used a combination of ∆CFI and ∆RMSEA [10] because the sample size did not fulfil the criteria for this fit-statistics which requires more than 300 subjects per group and equal in numbers across groups [41]. The Cronbach’s α values were satisfactorily within the recommended criteria of between 0.70 and 0.95 [27] for all subscales and were generally slightly higher than those of the previous study [10].

Discriminant validity by unpaired *t*-test of the Malay PIDAQ scores of participants with self-rated malocclusion (MI-S) and investigator-rated malocclusion (MI-D) showed statistically significant differences between those with no or slight malocclusion and those with severe malocclusion for all subscales, regardless of age. Those with severe malocclusion had lower DSC subscale mean scores and higher SI, PI and AC subscale mean scores than did those with slight malocclusion, which was reflected in the positive and negative signs of the effect sizes. This concurred with the study by Klages et al. [10]. Similar to the previous study [10], the strength of the effect sizes based on the MI-S were strongly above 0.80, but the effect sizes based on the MI-D were between medium (0.50) and strong (≥0.80) for each subscale.

Construct validity of the Malay PIDAQ was further assessed against ranking of perceived dental attractiveness and self-assessed need for dental correction while criterion validity was assessed against the impact of malocclusion on daily activities as assessed using the CS-OIDP with the following rationale. Since PIDAQ is concerned with the impact of dental attractiveness on the OHRQoL, it was reasonable to test its properties against participants’ self-perceived dental attractiveness. Those reporting an impact would likely also feel that they needed to have their teeth corrected, and if the impact affects their OHRQoL, the impact that is caused by malocclusion would most likely affect their daily activities. The results of the study concurred with these expectations. All subscales indeed showed statistically significant associations with perceived dental attractiveness rank, self-assessed need for dental correction and CS-OIDP, regardless of age. Those who felt they had excellent dental attractiveness had higher DSC subscale mean scores and lower SI, PI and AC subscale mean scores compared with those with lower self-rated dental attractiveness. Those who felt that they needed braces had lower DSC subscale mean scores and higher SI, PI and AC subscale mean scores compared with those who felt they did not need braces. Those with presence of CS-OIDP attributed to malocclusion also had lower DSC subscale mean scores and higher SI, PI and AC subscale mean scores than did those without CS-OIDP. This was further supported by a statistically significant negative correlation of the DSC subscale and positive correlation of the SI, PI and AC subscales with the performance scores of the CS-OIDP.

In terms of test-retest reliability, the ICC scores for all subscales were generally slightly lower, between 0.71 to 0.89, than those in the previous study, which were between 0.82 to 0.96 [10], but were all above the recommended minimum standard of 0.70 for reliability [27]. A statistically significant difference was detected in the PI subscale of the younger age group, but the difference was below the SDC score. In the study by Klages et al. [10], statistical significance was detected in the AC subscale of their younger age groups, but the differences were also below the SDC scores. The SDC reflects the smallest within-person change in score that can be interpreted as a real change provided that the difference is significant [27]. In this study, the SDC scores were higher than in the study by Klages et al. [10], indicating that higher score differences were needed in this population before the scores can be interpreted as true changes.

Presence of floor or ceiling effects reduces reliability since those with lowest and highest scores could not be distinguished from each other. Furthermore, the presence of these effects limits content validity since items in the extreme lower or upper part of the scale may be missing [27]. This study found no floor effects except in the AC subscale and no ceiling effects, since the prevalence of the lowest and highest possible scores were satisfactorily below the recommended maximum frequency of 15% [27]. The floor effects in the AC subscale were just above this value at 15.6% (12-17 years old age group) and 16.3% (12-14 years old age group). In Klages et al. [10], floor effects were present for the SI, PI and AC subscales. This demonstrates that the items of this instrument were sufficient to distinguish Malaysian adolescents with impact on their dental aesthetics at the lower and upper ends of the spectrum.

## Conclusion

Overall, the Malay PIDAQ has satisfactorily achieved the conceptual, item, semantic, operational and measurement equivalences similar to the original PIDAQ. While small modifications in the scale were required for this population, the Malay PIDAQ showed adequate validity and reliability to be used to assess the impact of malocclusion on the OHRQoL of Malaysian adolescents aged 12–17 years.

## Abbreviations

χ^2^: Chi-square
∆: Differences
AC: Aesthetic Concern
ANOVA: Analysis of variance
CFI: Comparative Fit Index
Child-OIDP: Child Oral Impacts on Daily Performance
CS-OIDP: Condition-specific Oral Impacts on Daily Performance
DSC: Dental Self - Confidence
ICC: Intraclass correlation coefficient
IOTN: Index of Orthodontic Treatment Need
IOTN-AC: Aesthetic Component of the Index of Orthodontic Treatment Need
IOTN-DHC: Dental Health Component of the Index of Orthodontic Treatment Need
MI-D: Malocclusion Index (Investigator-rated malocclusion)
MI-S: Malocclusion Index (Self-rated malocclusion)
OHRQoL: Oral Health Related Quality of Life
PI: Psychological Impact
PIDAQ: Psychosocial Impact of Dental Aesthetics
POS: Perception of Occlusion Scale
RMSEA: Root Mean Square Error of Approximation
SDC: Smallest detectable change
SEM: Standard error of measurement
SI: Social Impact
α: alpha
